# Anti-apoptotic and anti-fibrotic efficacy of exercise training in hypertensive hearts: A systematic review

**DOI:** 10.3389/fcvm.2023.1138705

**Published:** 2023-04-27

**Authors:** Adjar Yusrandi Akbar, Zhen-Yang Cui, Che-Jui Hsu, Yan-Zhang Li, Ferry Fadzlul Rahman, Chunqiu Xia, Ai-Lun Yang, Shin-Da Lee

**Affiliations:** ^1^ Department of Medical Laboratory Science and Biotechnology, Asia University, Taichung, Taiwan; ^2^ Department of Biology Education, University of Muhammadiyah Malang, Malang, Indonesia; ^3^ School of Rehabilitation Medicine, Weifang Medical University, Shandong, China; ^4^ Department of Physical Therapy, Graduate Institute of Rehabilitation Science, China Medical University, Taichung, Taiwan; ^5^ Department of Public Health, Universitas Muhammadiyah Kalimantan Timur, Kalimantan Timur, Indonesia; ^6^ College of Physical Education, Chengdu University, Chengdu, China; ^7^ Institute of Sports Sciences, University of Taipei, Taipei, Taiwan

**Keywords:** apoptosis, cardiac, exercise, fibrosis, hypertension, survival

## Abstract

**Background:**

This review aims to summarize the antiapoptotic, pro-survival, and antifibrotic effects of exercise training in hypertensive hearts.

**Methods:**

Keyword searches were conducted in PubMed, Web of Science, and Scopus in May 2021. Research published in English on the effects of exercise training on the apoptosis, survival, and fibrosis pathways in hypertension was included. The CAMARADES checklist was used to determine the quality of the studies. Two reviewers independently implemented predesigned protocols for the search and selection of studies, the assessment of study quality, and the evaluation of the strength of evidence.

**Results:**

Eleven studies were included after selection. The duration of the exercise training ranged from 5 to 27 weeks. Nine studies showed that exercise training improved cardiac survival rates by increasing IGF-1, IGF-1 receptor, p-PI3K, Bcl-2, HSP 72, and p-Akt. Furthermore, 10 studies showed that exercise training reduced apoptotic pathways by downregulating Bid, t-Bid, Bad, Bak, Bax, TNF, and FADD. Finally, two studies reported the modification and subsequent improvement of physiological characteristics of fibrosis and decreased MAPK p38 and PTEN levels by exercise training in the left ventricle of the heart.

**Conclusions:**

The findings of the review showed that exercise training could improve cardiac survival rates and attenuate cardiac apoptotic and fibrotic pathways in hypertension, suggesting that exercise training could act as a therapeutic approach to prevent hypertension-induced cardiac apoptosis and fibrosis.

**Systematic Review Registration:**

https://www.crd.york.ac.uk, identifier: CRD42021254118.

## 1. Introduction

Hypertension is regarded as the leading cause of cardiovascular disorders such as coronary heart disease, stroke, and heart failure and is responsible for 1.8 million deaths worldwide, with a projected increase to 23.3 million fatalities by 2030 (1–3). Hypertension is linked to the impaired diastolic function of the left ventricle, which is independent of the effect of obesity and other covariates (4). Previous research has established that animals and humans with arterial hypertension have abnormally high levels of cardiomyocyte apoptosis (5) and myocardial fibrosis (6). Furthermore, it can lead to cardiomyocyte loss in myocardial disease (7) and act as a predictor of adverse outcomes in heart failure (8).

Apoptosis is a physiological process of cell death (9), which is frequently induced either intrinsically or extrinsically via the death receptor–dependent apoptotic pathway (10, 11). In the initiation of the process, the apoptotic site starts from the mitochondrial membrane, where the proapoptotic component truncated Bid (t-Bid), the Bcl-2 family of proapoptotic components, the Bcl-2-associated death promoter (Bad), and the Bcl-2-associated X protein (Bax) can promote cytochrome-*c* activation into the cytosol, thereby activating caspase-9 and caspase-3 to generate the cell death process (10, 12, 13). Extrinsically, apoptosis begins with the binding of the Fas ligand or tumor necrosis factor (TNF) to the cell membrane. This is followed by the recruitment of the Fas-associated death domain (FADD), which upregulates the death-inducing signaling complex. Finally, it activates caspase-3 and caspase-8, triggering a cell death program (10). On the other hand, to promote cell survival, IGF-1 is reported to be the critical agent in modulating the survival response in cardiac tissue (14). The key signaling factors for IGF-1 and its receptor (IGF-1R) are highly dependent on protein kinase B (Akt) and phosphatidylinositol 3-kinase (PI3K). In mitochondria, activated Akt may have direct inhibitory effects on BAD (Bcl-2 antagonist of cell death), where Akt acts as an antiapoptototic component (15). Bcl-2 family members that are proapoptotic or pro-survival may affect and neutralize each other; therefore, the residual proteins determine the cytochrome-*c* release (14). Notably, Bcl-2 and Bcl-xL are involved in the activation of downstream apoptotic signaling, while BAD upregulates cytochrome-*c* release. Meanwhile, when BAD is phosphorylated (p-BAD), it becomes pro-survival, suppressing the release of cytochrome-*c* and apoptosis (16). When it is released, cytochrome-*c* enters the cytosol to activate caspase-9 and caspase-3, resulting in nucleosomal DNA breakage and execution of the apoptotic program. Therefore, apoptosis plays a significant role in the pathogenesis of various cardiac diseases; however, blocking the apoptotic pathway and targeting the survival pathway may help generate a better prognosis.

In fibrosis pathways, the signaling process is mediated by SMAD-dependent or SMAD-independent pathways and involves TGF-β. In the SMAD-dependent process, TGF-β promotes the formation of complex heterotetrameric molecules that activate SMAD2 and SMAD3, thus dissociating them from receptors and the complex with SMAD4 in the cytosol (17). This SMAD complex migrates to the nucleus as a transcription factor (18). In fibroblasts, the SMAD-dependent pathway causes myofibroblast differentiation and produces increased levels of profibrotic genes such as periostin, collagen, and smooth muscle actin (19). In the SMAD-independent pathway, a similar profibrotic effect is produced in fibroblasts, and there is crosstalk between these two pathways. This response is induced by mitogen-activated protein kinase (MAPK), including ERK, p38, and JNK (20). As a result, the mechanical and physical properties of the environment are altered, thus producing excessive collagen volume in the tissue. In cardiac rehabilitation, exercise training has significantly improved the prevention and treatment of cardiovascular disease (21, 22). Exercise is one of the non-pharmacological therapies for chronic heart disease, and it helps patients improve their exercise capacity and quality of life (23). It also protects against increased apoptosis, according to Kwak et al. (24), by reducing caspase-9 levels and the Bax/Bcl-2 ratio. In addition, in hypertensive conditions, exercise training reduces resting heart rate and, as a result, blood pressure (16, 25), increases capillary growth (26), and lowers myocardial oxygen consumption for a given workload (27). Besides, it inhibits the abnormal proliferation of mitochondria and myofibrils that characterize left ventricular hypertrophy by ensuring sufficient mitochondrial development (28).

These data suggest that exercise training may act as a cardioprotective shield against hypertension. However, the existence of a large number of approaches in each study makes it difficult to fully understand the effects of exercise training on the apoptosis, survival, and fibrosis pathways at the molecular level. Although one systematic review discussed the benefit of exercise in promoting cardioprotection, the area of discussion did not specifically focus on the apoptosis, survival, and fibrosis pathways in hypertension. This systematic review aims to fill this gap and was conducted to summarize the effects of exercise training on these three pathways in hypertension.

## 2. Materials and methods

### 2.1. Eligibility criteria

#### 2.1.1. Types of study designs

We included only English-language studies with controlled trials and separate experimental groups, regardless of publication date. We excluded case reports, protocol articles, reviews, or conference abstracts.

#### 2.1.2. Types of animal models

We included any type of hypertension model, and their age, species, and sex were not restricted. Reports were excluded if they did not provide sufficient information about the hypertension model. Furthermore, *in vitro* and *in situ* models were excluded.

#### 2.1.3. Types of intervention

We included exercise training such as swimming, treadmill running, and wheel running without any protocol restrictions. Information on exercise type, or speed had to be provided. We excluded studies that examined the effects of exercise training in combination with other therapies or acute exercise training.

#### 2.1.4. Types of comparators

We included trials that compared non-exercise control, sedentary, and normotensive groups.

#### 2.1.5. Types of outcomes

The effects of exercise training on the apoptosis pathway are determined by the expression of Bak, Bad, Bax, t-Bid, Bid, FADD, TNF receptor 1, TNF, Fas, Fas ligand, cytochrome-*c*, and caspase, while the survival pathway is determined by the expression of IGF-1, IGF-1R, PI3K, Akt, p-Bad, HSP-72, and Bcl2. Finally, the fibrosis pathway is determined by the expression of TGF-β, MAPK, MMPs, and CTGF.

### 2.2. Information sources and search strategy

**Figure 1:**
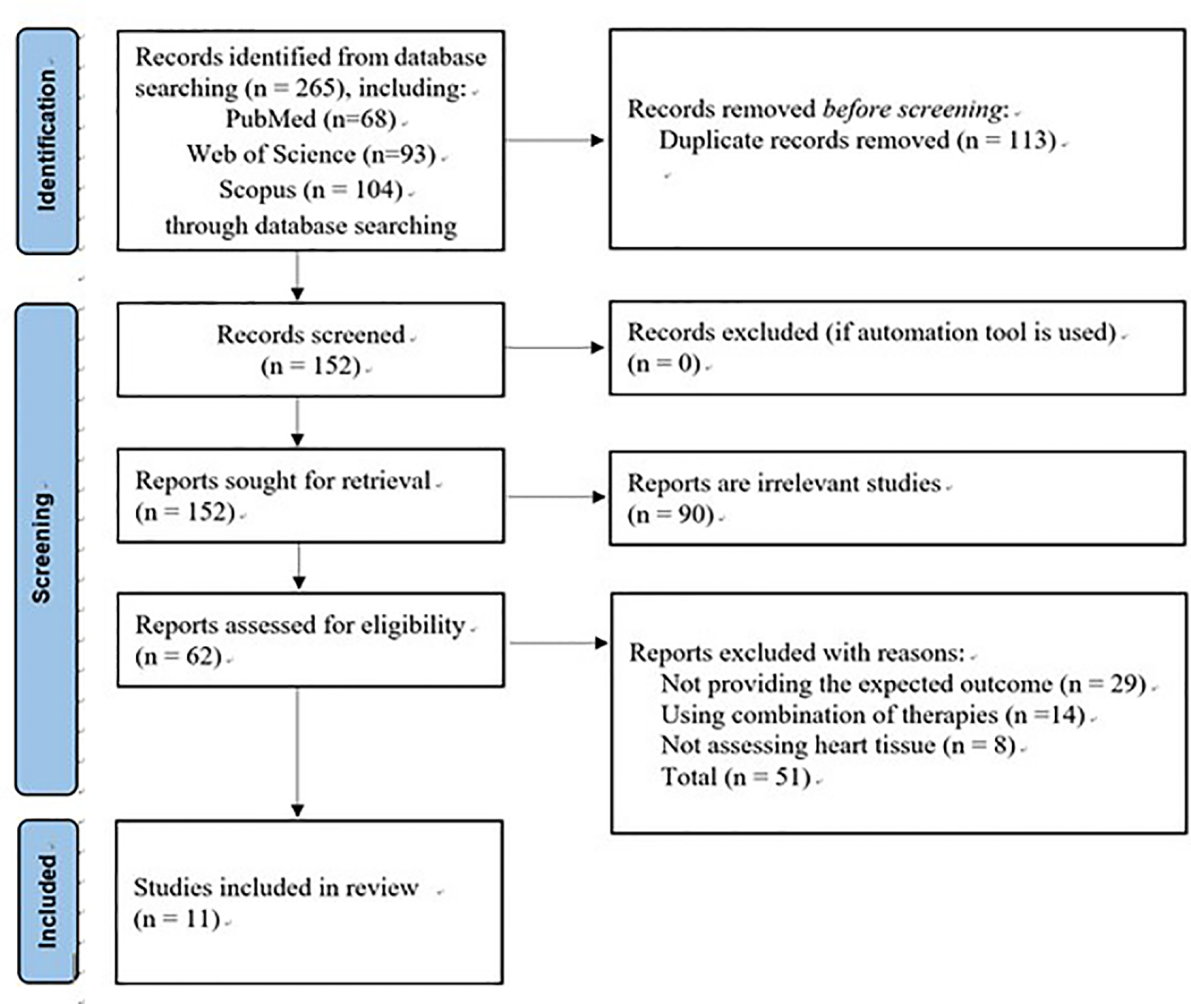
PRISMA flow diagram (2020) for the selected protocol.

In May 2021, relevant studies were identified by keyword searches in the PubMed, Web of Science, and Scopus databases, with a combination of the following terms: (“*Rats*” OR “*SHR*”) AND (“*Hypertensive*” OR “*Hypertension*”) AND (“*Training*” OR “*Exercise*”) AND (“*Heart*” OR “*Cardiac*”) AND (“*Anti-apoptotic*” OR “*Apoptosis”* OR “*Fibrosis*” OR “*Bax*” OR “*Bcl-2*” OR “*Tbid*” OR “Caspase”). We retrieved title and abstract keywords from the databases using the search strategy and reviewed the references of the included studies to identify additional eligible papers. To elaborate, the titles and abstracts of the studies were screened to eliminate duplicates and irrelevant studies that did not include the apoptosis, survival, and fibrosis pathways and exercise training information in their abstracts. Full texts were then assessed and read using inclusion and exclusion criteria to select studies. Two independent reviewers conducted the study selection process. Whenever disagreements arose, they conferred with a third consultant to reach a consensus (Figure 1).

### 2.3. Selection process

The two authors worked independently to select the included studies based on the inclusion criteria. However, any disagreements were resolved by the third consultant. Furthermore, no automated tools were used in the selection process.

### 2.4. Data collection process

Two independent reviewers extracted data from the included articles by reading the text, graphs, and tables (including study characteristics and outcomes). If the data were not readily available, we contacted the authors to obtain them. For study characteristics, we retrieved the first author's name, year of publication, hypertension model (type, species, and sex), and exercise technique (type, timing, frequency, duration, or speed). For outcome extraction, we focused on the effect of exercise training on the apoptotic, survival, and fibrosis pathways in hypertension.

### 2.5. Study of the risk of bias in assessment

The 10-item “Collaborative Approach to Meta-Analysis and Review of Animal Data from Experimental Studies” (CAMARADES) checklist was used to assess the quality of the study. The two authors separately examined and completed the predesigned CARAMADES checklist datasheets. Any disputes or disagreements were resolved through discussions between the two authors and the third consultant.

### 2.6. Effects measures

The effect measures of the apoptosis, survival, and fibrosis pathways did not change, increase, or decrease the protein levels in the experimental group compared to the control group.

### 2.7. Data synthesis and presentation

The PRISMA flowchart and narrative synthesis presented the results of the search strategy. The characteristics of the study and its outcomes were presented in text and tables. A summary of the exercise training (type, timing, frequency, duration, or speed) and apoptosis, survival, and fibrosis pathways in hypertension was provided to determine the study characteristics. For the outcomes, the effects of exercise training on the apoptosis and survival pathways were described by considering the protein expression level, while fibrosis was described on the basis of its percentage in hypertension. The results were described by comparing the non-exercise control and sedentary groups.

### 2.8. Reporting bias assessment

The CAMARADES checklist was used to examine the possibility of bias in the research. The text and tables show the list of authors and the rubric. The following are the ten items on the rubric for evaluating bias in each paper: (1) Publication in a peer-reviewed journal, (2) Temperature control statement, (3) Randomization of control or treatment, (4) concealment of allocation, (5) Blinded evaluation of outcome, (6) Avoidance of anesthetics with distinct intrinsic properties, (7) Use of a hypertension model, (8) Calculation of sample size, (9) Statement of compliance with regulatory standards, (10) Statement of potential conflict of interest.

## 3. Results

The results of the search strategy showed that articles were found in PubMed (*n* = 68), Web of Science (*n* = 93), and Scopus (*n* = 104). After screening the title and abstract, we removed duplicate (*n* = 113) and irrelevant (*n* = 90) studies. Furthermore, a full-text review was performed. We excluded 51 studies: 29 studies with irrelevant outcomes, 14 that contained references to combination therapies, and 8 that did not have cardiac tissue assessment. Finally, 11 studies were included in the systematic review for analysis.

### 3.1. Types of hypertension models

Four hypertension models were used in the included studies, namely, the spontaneously hypertensive rat (SHR) model (*n* = 8), the spontaneously hypertensive heart failure rat (SHHF) model (*n* = 1), the Dahl salt-sensitive (DS) male rat model (*n* = 1), and the monocrotaline model (*n* = 1) (Table 1).

**Table 1:** Characteristics of included studies and summary data.

Author	Model	Exercise training	Heart's tissue	Outcomes and measurement method				
				Survival pathway	Apoptosis pathway		Fibrosis pathway	
					Mitochondria-dependent pathway	Fas-dependent pathway	Fibrosis components	Fibrosis physiological characteristic
1. Huang et al. (16)	SHR (8 weeks old)	Treadmill protocol • Duration: 60 min, 5 days/week, 12 weeks • Speed: 27 m/min	Left ventricle	**Measurement** Western blotting (**HTN**): IGF-1, IGF-1 receptor, p-PI3K, and p-Akt were reduced; (**EX**): IGF-1, IGF-1 receptor, p-PI3K, and p-Akt were upregulated	**Measurement** Western blotting (**HTN**): Increases in t-Bid, Bad, Bak, Bax, and cytochrome-*c*; Increases in caspase-8, caspase-9, and caspase-3; (**EX**): Decreases in t-Bid, Bad, Bak, Bax, and cytochrome-*c*; Decreases in caspase-8, caspase-9, and caspase-3	**Measurement** Western blotting (**HTN**): Increases in the Fas ligand, Fas, TNF, TNF receptor 1, and FADD; (**EX**): Decreases in the Fas ligand, Fas, TNF, TNF receptor 1, and FADD	**Measurement** Western blotting (**HTN**): Increase in PTEN levels; (**EX**): PTEN was downregulated	**Measurement** Masson’s trichrome staining (**HTN**): Cardiac fibrosis percentage was increased; (**EX**): Decrease in cardiac fibrosis
2. Lee et al. (29)	SHR (6 weeks old)	Treadmill protocol • Duration: 60 min, 5 days/week, 13 weeks • Speed: 20 m/min	Left ventricle	**Measurement** Western blotting (**HTN**): Bcl-2 was decreased; (**EX**): Bcl-2 was increased	**Measurement** Western blotting (**HTN**): Increases in Bax and caspase-3; (**EX**): Bax and caspase-3 were downregulated	N/A	N/A	**Measurement** Manual weight and length measurement (**HTN**): Increased wall thickness of the left ventricle; (**EX**): Wall thickness was reduced
3. Libonati et al. (30)	SHR (4 months old)	Treadmill protocol • Duration: 60 min, 5 days/week, 12 weeks • Speed: 25 m/min	Left ventricle	**Measurement** Western blotting (**HTN**): Decrease in p-Akt levels; (**EX**): p-Akt was not changed	**Measurement** Western blotting (**HTN**): Bad, caspase-3, and caspase-9 were upregulated; (**EX**): Increase in caspase-3; Bad and caspase 9 levels unchanged	N/A	N/A	
4. Watson et al. (31)	SHHF (7 months old)	Treadmill protocol • Duration: 45 min, 3 days/week, 6 months • Speed: 14 m/min	Left ventricle	**Measurement** Western blotting (**HTN**): Decreased in Bcl-2; (**EX**): Bcl-2 was upregulated	**Measurement** Western blotting (**HTN**): Increases in Bax, caspase-3, and cytochrome-3; (**EX**): Bax, caspase-3, and cytochrome-3 were downregulated	N/A	N/A	
5. Lin et al. (32)	SHR (15 weeks old)	Treadmill protocol • Duration: 60 min, 5 days/week, 8 weeks • Speed: 15–27 m/min	Left ventricle	**Measurement** Western blotting (**HTN**): Decreased in p-PI3K, p-Akt, and Bcl-2; (**EX**): Increased in PI3K, p-Akt, and Bcl-2 levels	**Measurement** Western blotting (**HTN**): t-Bid, Bad, Bak, Bax, cytochrome-*c*, activated caspase-9 and caspase-3 were upregulated; (**EX**): Decreases in t-Bid, Bad, Bak, Bax, cytochrome-*c*, activated caspase-9 and caspase-3	**Measurement** Western blotting (**HTN**): Increases in Fas ligand, TNF-α, Fas receptors, FADD and activated caspase-8; (**EX**): Decreases in Fas ligand, TNF-α, Fas receptors, FADD and activated caspase-8	N/A	**Measurement** Masson’s trichrome staining **(HTN**): The percentage of cardiac fibrosis was increased; (**EX**): Decreased cardiac fibrosis
6. McMillan et al. (33)	SHR (11 weeks old)	Treadmill protocol • Duration: 45 min, 5 days/week, 6 weeks • Speed: 21 m/min	Left ventricle	**Measurement** Western blotting (**HTN**): Decreased level of p-Akt; (**EX**): The levels of p-Akt was upregulated	**Measurement** Western blotting (**HTN**): Increases in caspase-3 and calpain; (**EX**): Decreases in caspase-3 and calpain	N/A	N/A	
7. Lajoie et al. (34)	SHR (3 weeks old)	Treadmill protocol • Duration: 120 min, 5 days/week, 8 weeks • Speed: 15–18 m/min	Left ventricle	**Measurement** Western blotting (**HTN**): Decreased levels of Bcl 2 and HSP 72; (**EX**): The levels of Bcl-2 and HSP 72 were upregulated	**Measurement** Western blotting (**HTN**): Increase in Bax; (**EX**): Bax was upregulated	N/A	N/A	
8. Miyachi et al. (35)	Male Dahl salt-sensitive (DS) rats (9 weeks old)	Swimming Protocol after NaCl administration • Duration: 60 min, 5 days/week, 9 weeks	Left ventricle	**Measurement** Immunoblot (**HTN**): Decreased levels of PI3K and p-Akt; (**EX**): Increased levels of PI3K and p-Akt	N/A	N/A	**Measurement** Immunoblot (**HTN**): Increase in p38 MAPK; (**EX**): Decrease in p38 MAPK	**Measurement** Echocardiograph **(HTN**): The percentage of cardiac fibrosis was increased; (**EX**): Decreased cardiac fibrosis
9. Kolwicz et al. (36)	SHR (16 weeks old)	Treadmill protocol • Duration: 60 min, 5 days/week, 12 weeks • Speed: 20–25 m/min	Left ventricle	**Measurement** Western blotting (**HTN**): Decreased level of Akt; (**EX**): Akt level was not changed	**Measurement** Western blotting (**HTN**): Increases in the apoptotic index (TUNEL nuclei divided by the total number of sampled cardiomyocyte nuclei); (**EX**): Decreases in the apoptotic index	N/A	N/A	
10. Colombo et al. (37)	Male Wistar rats 4 weeks old + pulmonary hypertension	Treadmill after monocrotaline administration • Duration: 60 min, 5 days/week, 5 weeks • Speed: 0.6–0.9 km/hour (10–15 m/min)	Right ventricle	**Measurement** Western blotting (**HTN**): Decreased Akt and PI3K levels; (**EX**): Increased levels of Akt and PI3K	**Measurement** Western blotting (**HTN**): Increases in caspase-3 and Bax to Bcl 2 ratio; (**EX**): caspase-3 was decreased; Bax to Bcl-2 ratio unchanged	N/A	N/A	
11. Garciarena et al. (38)	SHR (4 months old)	Swimming protocol • Duration: 90 min, 5 days/week, 60 days	Left ventricle	**Measurement** Western blotting (**HTN**): Decreased PI3K and Akt levels; (**EX**): PI3K and Akt levels unchanged	**Measurement** Western blotting (**HTN**): Increase in caspase-3; (**EX**): Caspase-3 was downregulated	N/A	N/A	**Measurement** Masson’s trichrome staining **(HTN**): Collagen volume fraction in left ventricle was increased; (**EX**): Reduced collagen volume fraction

SHR, spontaneously hypertensive rats; SHHF, spontaneously hypertensive heart failure rats; (HTN), HTN relative to the control group; (EX), exercise in HTN relative to SHR group; IGF-1, insulin-like growth hormone; p-PI3K, phosphatidylinositol 3-kinase; Akt, protein kinase B; p-Akt, phosphorylated Akt; Bcl-2, B-cell lymphoma-2; HSP 72, heat shock protein 72; Bid, BH3-interacting domain death; t-Bid, truncated Bid; Bad, Bcl-2-associated death; Bak, Bcl-2 homologous antagonist killer; Bax, Bcl-2-associated X protein; TNF, tumor necrosis factor; FADD, fas-associated death domain protein; N/A, not available.

The table shows the characteristics of selected studies and outcomes. The characteristics of the studies included the name of the first author, hypertension model, exercise training, and the areas of cardiac tissue analyzed. The outcomes are the survival pathway, apoptosis pathway, and fibrosis pathway. All the outcomes were described in the comparison among the non-exercise SHR control group, the sedentary SHR group, and the exercise-trained SHR group.

### 3.2. Types of exercise training

There were two types of exercise training: treadmill exercise (*n* = 9) and swimming exercise (*n* = 2). In the former, the daily duration ranged from 45 to 120 min for 3–5 days/week. The treadmill speed ranged from 10 m/min to 27 m/min. In addition, there were periods of 5–27 weeks. In the latter (swimming exercise), the duration ranged from 60 to 90 min/day for five days/week. The duration of swimming also ranged from 8 to 9 weeks.

### 3.3. Types of outcomes

The included studies analyzed 11 articles in which left ventricular tissue was observed. Measurement methods were determined by using Western blot analysis (*n* = 10) (16, 29–34, 36–38) and immunoblot (*n* = 1) (35) for survival pathways. All measurements of the apoptosis pathway studies (*n* = 10) (16, 29–34, 36–38) were performed by Western blot. Meanwhile, for the fibrosis pathway analysis, Masson’s trichrome staining was used to conduct a histopathological analysis (*n* = 3) (16, 32, 38), manual measurements for wall thickness (*n* = 1) (29), and echocardiography for the interstitial fibrosis analysis (*n* = 1) (35). For the survival pathway, nine studies reported an alteration in the cell survival pathway, including IGF-1, IGF-1 receptor, p-PI3K, Bcl-2, HSP 72, and p-Akt. For the apoptosis pathway, 10 studies evaluated the levels of apoptosis proteins, such as BH3-interacting domain death (Bid), truncated Bid (t-Bid), Bcl-2-associated death (Bad), Bcl-2 homologous antagonist killer (Bak), Bcl-2-associated X protein (Bax) (mitochondrial pathway), tumor necrosis factor (TNF), and Fas-associated death domain protein (FADD) (Fas pathway). However, studies (39, 40) that failed to provide details of the apoptosis protein profile were excluded from this study. In addition, five studies assessed cardiac fibrosis, wall thickness, and collagen volume fraction in the left ventricle for the fibrosis pathway.

### 3.4. Effects of exercise training on the survival pathway in hypertension

Four studies analyzed the effect of exercise training on Bcl-2 in hypertension and showed similar results (29, 31, 32, 34). These studies indicated that the level of Bcl-2 was decreased in the sedentary SHR group (31, 32, 34) and was normal in the SHR group (29), whereas exercise training increased the level of hypertension.

Eight studies analyzed the effect of exercise training on Akt/p-Akt in hypertension and reported different results. Five studies reported that Akt/p-Akt levels were decreased in hypertension compared with the sedentary SHR group, whereas exercise training increased these levels in hypertension (16, 32, 33, 35, 37). Also, one study showed that the Akt/p-Akt levels were unchanged in the sedentary and exercise-trained SHR groups (30). However, two studies reported contrasting results in which Akt/p-Akt levels were reduced in the sedentary SHR group, and exercise training decreased these levels (36, 38).

Five studies analyzed the effect of exercise training on PI3K/p-PI3K levels in hypertension. Four studies reported that these levels were reduced in hypertension compared with the sedentary SHR and non-exercise SHR groups, whereas exercise training restored these levels in hypertension (16, 32, 35, 37). In contrast, one study reported that PI3K/p-PI3K levels were reduced in the sedentary SHR and non-exercise SHR groups, whereas exercise training decreased these levels in hypertension (38).

One study observed that the protein level of IGF-1 was reduced in the non-exercise SHR group, whereas exercise training increased its level of hypertension (16). Also, one study reported that the expression of HSP 72 was reduced in the sedentary SHR group, and exercise training restored its level of hypertension (34).

### 3.5. Effects of exercise training on the apoptosis pathway in hypertension

Seven studies reported the effect of exercise training on the protein levels of t-Bid, Bad, Bak, and Bax in hypertension and reported different results. Four studies reported that the levels of t-Bid, Bad, Bak, and Bax were increased in the sedentary SHR group and the non-exercise group, whereas exercise training reduced these levels of hypertension (16, 29, 31, 32). In addition, two studies showed that the protein levels of Bax and Bad were unchanged in the sedentary SHR group and exercise-trained SHR group (30, 37). Finally, one study reported opposite results, showing that the protein level of Bax was increased in the sedentary SHR group and that exercise training increased this level of hypertension (34).

Eight studies reported the effect of exercise training on caspase levels in hypertension. Seven studies observed that the levels of caspase-3 (16, 29, 31–34, 38), caspase-8 (16), and caspase-9 (16, 32) were increased in the sedentary SHR and non-exercise SHR groups, whereas exercise training reduced these levels. However, one study reported that the caspase-9 level remained unchanged in the sedentary SHR and the exercise-trained SHR groups (30). This study also showed that the level of caspase-3 was increased in the sedentary SHR group, whereas exercise training increased its level (30).

Three authors observed the effect of exercise training on cytochrome-*c* levels in hypertension. These reports (16, 31, 32) showed that this level was increased in the sedentary SHR group, whereas exercise training decreased its level. Furthermore, two studies reported that the levels of the Fas ligand, TNF, and FADD were increased in the sedentary SHR and non-exercise SHR groups, whereas exercise training lowered these levels (16, 32).

### 3.6. Effects of exercise training on the fibrosis pathway in hypertension

Five studies reported the effect of exercise training on the fibrosis pathway in hypertension. Three authors observed that the degree of cardiac fibrosis was increased in the sedentary SHR and non-exercise SHR groups, whereas exercise training attenuated its extent (16, 32, 35). Notably, two studies reported that MAPK p38 and PTEN levels were elevated in the SHR group, whereas exercise training reduced them (16, 35). Furthermore, one study reported that the left ventricular wall thickness was increased in the non-exercise SHR group, whereas exercise training reduced its thickness (29). Finally, one study reported an increased collagen volume fraction in the sedentary SHR group, whereas exercise training reduced the volume (38).

### 3.7. Risk of bias in the studies

All of these studies were published in peer-reviewed publications with randomization of treatment controls and a statement of regulatory compliance. However, there was no evidence in the research of allocation concealment, blinded assessment, or sample size calculation. Three studies (27.3%) did not provide clear explanations of the anesthetic procedures, and six studies (54.5%) did not include a conflict of interest statement. Moreover, five reports (45.4%) lacked information on temperature control. According to the CAMARADES criteria, the overall median score was 6 (Table 2).

**Table 2:** CAMARADES study quality checklist.

No	Author	CAMARADES study quality checklist										
		1	2	3	4	5	6	7	8	9	10	Total
1	Huang et al. (16)	✓	✓	✓				✓		✓	✓	6
2	Lee et al. (29)	✓	✓	✓			✓	✓		✓	✓	7
3	Libonati et al. (30)	✓		✓			✓	✓		✓	✓	6
4	Watson et al. (31)	✓		✓			✓	✓		✓		5
5	Lin et al. (32)	✓	✓	✓			✓	✓		✓	✓	7
6	McMillan et al. (33)	✓		✓				✓		✓		4
7	Lajoie et al. (34)	✓		✓			✓	✓		✓		5
8	Miyachi et al. (35)	✓	✓	✓			✓	✓		✓		6
9	Kolwicz et al. (36)	✓		✓				✓		✓		4
10	Colombo et al. (37)	✓	✓	✓			✓	✓		✓	✓	7
11	Garciarena et al. (38)	✓	✓	✓			✓	✓		✓		6

(1) Publication in a peer-reviewed journal, (2) statement of temperature control, (3) randomization to treatment or control, (4) allocation concealment, (5) blinded assessment of outcome, (6) avoidance of anesthetics with marked intrinsic properties, (7) use of a hypertension model, (8) sample size calculation, (9) statement of compliance with regulatory requirements, and (10) statement of potential conflict of interest.

## 4. Discussion

**Figure 2:**
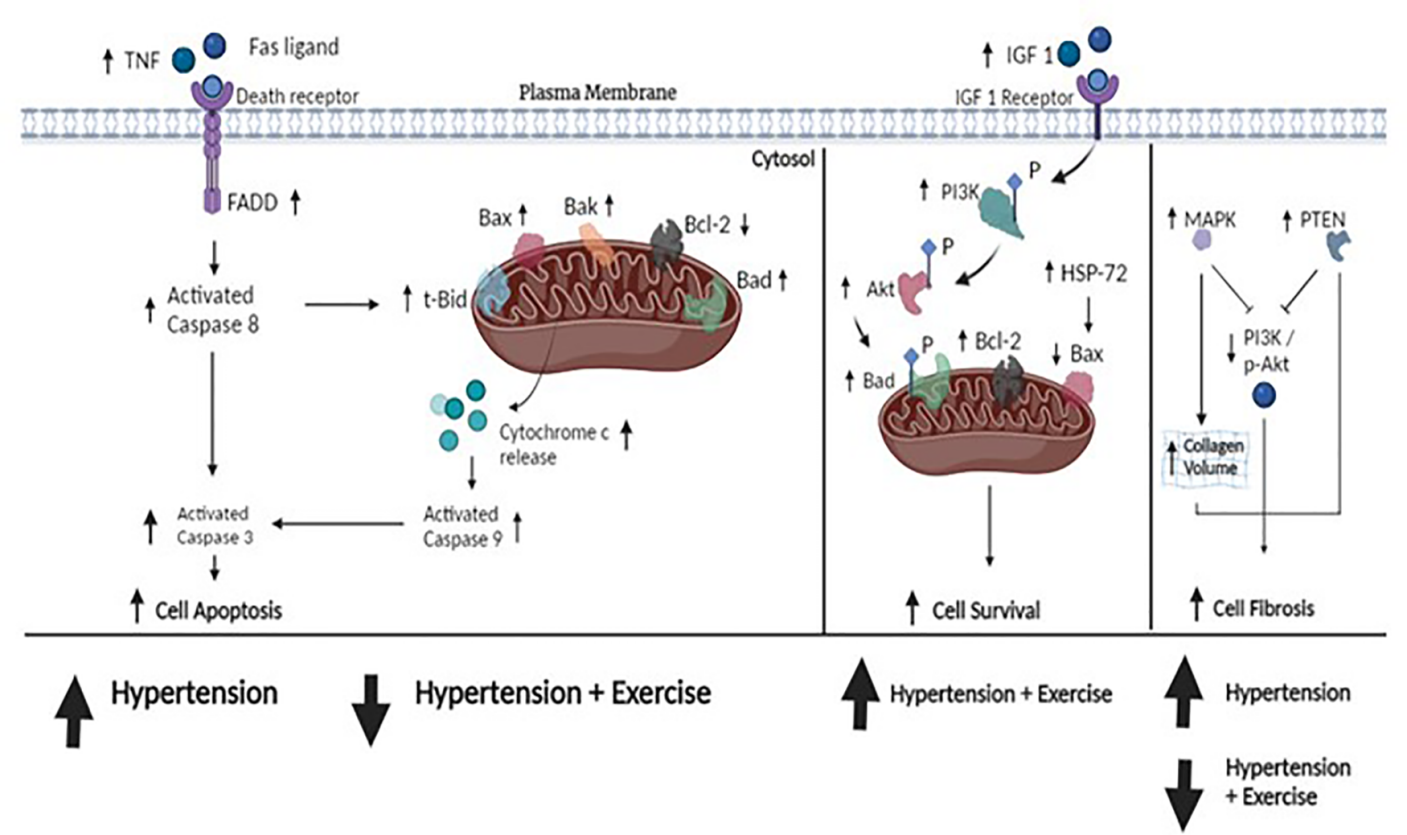
The hypothesized figure. The figure shows the pathway of cell apoptosis, cell survival, and cell fibrosis. Upstream, TNF plays an initial role in cell apoptosis by binding to death receptors in the cell membrane. This is followed by the recruitment of the Fas-associated death domain (FADD), resulting in the development of a death-inducing signaling complex that activates downstream caspase-8 and directs caspase-3 cleavage. Eventually, caspase-3 activation leads to cell apoptosis. Downstream, caspase-8 can directly elevate the proapoptotic protein t-Bid, which releases cytochrome-c into the cytosol, thereby activating caspase-9 and also directly cleaving caspase-3 as well. Other elevated proapoptotic proteins (e.g., Bad, Bax, and Bak) could induce apoptosis. In hypertension, the evidence showed that these proapoptotic proteins were overexpressed. The included studies suggested that exercise training could attenuate the expression of proapoptotic protein production. Furthermore, cell survival is controlled by an alteration of antiapoptotic proteins (IGF 1, IGF receptor, PI3K, Akt, Bcl-2, and HSP-72). PI3K and Akt are the keys to promoting cell survival by forming p-Bad and increasing Bcl-2 in mitochondria, thus preventing apoptosis. Our included studies suggested that hypertension treated by exercise training could upregulate the expression of cell survival. In cell fibrosis, the expression of MAPK p38 leads to increased collagen volume in the heart and the upregulation of PTEN inhibits the p-Akt/PI3K activity, thereby increasing cell fibrosis. Similarly, exercise training reduced both PTEN and MAPK p38 activity. Taken together, our included studies demonstrated that hypertension altered these protein expressions and consequently damaged them, whereas exercise training could repair them. These data suggest that exercise training could attenuate cell apoptosis, increase cell survival, and repair fibrosis in cardiac tissue under the condition of hypertension.

The results of the systematic review can be described as follows: (1) Exercise training could help enhance cardiac survival rates in hypertension by increasing the protein levels of Bcl-2, Akt, p-Akt, PI3K, IGF-1, and HSP 72. (2) Exercise training could help improve cardiac apoptosis by decreasing the protein levels of t-Bid, Bad, Bak, Bax, caspase, and cytochrome-c (mitochondrial pathway) and lowering the protein levels of the Fas ligand, TNF, TNF receptor 1, and FADD (Fas pathway) in hypertension. (3) Exercise training resolved the fibrosis in the left ventricle, which is supported by the evidence that exercise reduced MAPK p38 and PTEN levels and altered and consequently improved physiological characteristics by restoring cardiac fibrosis percentage, wall thickness, and collagen volume fraction in hypertension. Therefore, based on these findings and those that previously described the pathophysiology of hypertension together, we propose a hypothesis, as shown in Figure 2, which suggests that exercise training could help counteract the damage to cardiac tissue in the condition of hypertension. This mechanism is implemented by enhancing cell survival ability, suppressing apoptosis activity, and resolving cardiac fibrosis. This suggests that exercise training may have a therapeutic effect on heart tissue in hypertension.

### 4.1. Effects of exercise training on cardiac survival in hypertension

Based on the above findings, cardiac survival rates in hypertension were defined in terms of Bcl-2, Akt, p-Akt, PI3K, p-PI3K, IGF-1, and HSP 72. Furthermore, the included studies reported that exercise training increased the levels of Bcl-2, Akt, p-Akt, PI3K, p-PI3K, IGF-1, and HSP 72 cardiac tissue in hypertension (16, 29, 32–35, 37), suggesting that exercise training increases the chances of cell survival in the cardiac tissue to counteract hypertension. Previous research suggested that swimming for 60 min, 5 days/week, helped restore IGF-1 receptor, Akt, and pro-survival Bcl-2 protein levels in aging rats (14). Hypertension causes the suppression of IGF-1 signaling, whereas exercise training elevates IGF-1 levels and promotes the activation of PI3K and Akt signaling. PI3K/Akt signaling prevents apoptosis by mediating IGF-1 to express Bcl-2 and promoting BAD phosphorylation. As a result, antiapoptotic Bcl-2 levels significantly increase and promote cell survival. Furthermore, exercise training also enhances the protein level of heat shock protein 72 (HSP 72) in hypertension, which prevents apoptosis by inhibiting the release of cytochrome-*c* into the cytosol. Downstream of the mitochondrial pathway, HSP 72 contributes to maintaining the overexpression of the Bax protein and interferes with the activation of caspase-9. Thus, exercise training prevents apoptosis in hypertensive cardiac tissue by interfering with the proapoptotic proteins and caspase activation, resulting in cell survival.

However, among the included studies, three reports showed different results, whereas Akt levels did not change (30) and even decreased in the exercise training group (36, 38). Despite the data showing that Akt/PI3K expressions were similar in the sedentary hypertensive and exercise training hypertensive groups, exercise training still increased BAD phosphorylation (30). The plausible explanation is that calcineurin was reported to be decreased in the exercise hypertensive group compared to the sedentary group in the same study. Calcineurin mediates BAD dephosphorylation by binding to Bcl-x (L), resulting in caspase-3 activation. Consequently, the lower level of calcineurin due to exercise training did not translate into caspase-3 activation in the downstream regulation. Thus, the survival mechanism may be different and become more complex because it does not rely on specific protein signaling processes.

### 4.2. Effects of exercise training on cardiac apoptosis in hypertension

In the regulation of apoptosis, exercise training downregulated the proapoptotic protein in hypertension by decreasing the upstream regulation of apoptosis such as the Fas ligand, TNF, TNF receptor 1, and FADD, and the downstream regulation of apoptosis such as t-Bid, Bad, Bak, and Bax (16, 29, 31–33, 37, 38). Another study suggested that 13 weeks of treadmill exercise inhibited apoptosis by downregulating the Bad protein in isoproterenol rats (41). Furthermore, a study by Lee et al. explained that 3 months of treadmill exercise could help counteract the apoptotic Fas pathway by lowering TNF-α, Fas ligand, Fas receptors, and FADD, thus preventing caspase-8 and caspase-3 activation in obese rats (42). Also, the proapoptotic protein levels of Bad and Bax were restored by treadmill exercise, thus preventing caspase-9 and caspase-3 activation (42). Therefore, the results of our systematic review are consistent with those of previous studies that dealt with exercise-induced cardioprotective pathways. Overall, we hypothesized that exercise training could reduce myocardial apoptosis in the cardiovascular system in hypertensive disease.

With regard to the apoptosis pathway, the three included studies reported different results. Two studies showed that the levels of Bad, Bax, caspase-3, and caspase-9 remained unchanged in the exercise SHR group (30, 37). However, one study reported the opposite, showing that the level of Bax was increased in the exercise SHR group (34). The plausible explanation for such a discrepancy is the increased workload due to elevated blood pressure and lower glycogen content after 120 min of exercise. Glycogen content plays a role in glucose production, whereas previous studies have suggested that glucose limitation leads to Bax translocation (43). Normally, myocardial glycogen content is restored after 2–8 h of recovery (44); however, in SHR rats, the mechanism of glycogen restoration remains unknown. In addition, the SHR heart is considered to be insulin-resistant and impaired in fatty acid oxidation, which leads to a greater dependence on glycogen content (45).

### 4.3. Effects of exercise training on cardiac fibrosis in hypertension

In addition, our review reported that exercise training could repair the fibrosis pathway in cardiac tissue in hypertensive disease. The included studies showed that hypertension elevated the levels of MAPK p38 and PTEN and damaged the physiological characteristics, which resulted in a higher percentage of cardiac fibrosis, increased left ventricular wall thickness, and increased collenge percentage, whereas exercise training helped reduce the levels and repair the characteristics (16, 29, 32, 35, 38).

Our included studies analyzed only limited data on proteins involved in the fibrosis pathway; however, collagen volume alteration in the heart tissue was observed. To justify our result, a previous study in diabetic rats demonstrated that 8-week moderate exercise training downregulated TGF-β1, Smad, and MMP expressions, which reduced myocardial fibrosis in the heart tissue (46). Thus, we suggest that the change in collagen volume indicates that the process of fibrosis pathway regulation occurs. Also, another study showed that 9 weeks of treadmill exercise caused significant changes in myocardial architecture and reduced fibrosis in doxorubicin-treated rats (47). The preservation of myofibrillar integrity and sarcomere organization was more pronounced in the exercise-Dox group and was likely correlated with the reduction of oxidative stress, and exercise increased cardiac antioxidant defenses (47), thereby increasing the levels of antioxidant enzymes. Similarly, exercise training could help to decrease cardiac remodeling and reduce interstitial myocardial fibrosis in ovariectomized rats (48). Estrogen deprivation in ovariectomy promotes cardiomyopathic changes by increasing left ventricular weight, causing abnormal myocardial organization, enlarging the interstitial space, and promoting cardiac fibrosis, whereas 10 weeks of treadmill running could help repair fibrotic damage (49). Thus, we hypothesized that exercise training could repair the fibrosis pathway in cardiac tissue in hypertension.

In contrast, the established studies demonstrated different outcomes where IGF and Akt signaling contribute to fibrosis during cardiac remodeling. Zhao et al. observed that Akt activation in human cardiac fibroblasts (HCF) and rats (C57BL/6) caused myocardial fibrosis via the myocardial infarction–associated transcript (MIAT), resulting in increased IL-1β, IL-6, and TNF-α mRNA levels and numerous proteins that collectively contribute to the onset of heart failure (50). Also, prolonged activation of Akt induces pathological hypertrophy and heart failure by increasing angiogenesis in a disorganized manner, which is reminiscent of tumor vasculature (51). Nonetheless, beneficial results have been observed in exercise training interventions where Akt and IGF increases have been analyzed in hypertension (35, 52). Exercise training has been shown to reverse cardiac fibrosis by promoting the expression of antifibrotic factors and increasing the activity of matrix metalloproteinases that degrade excess collagen (53). Moreover, exercise training has been proven to promote vasodilation, which improves cardiac function and reduces the hypertensive load on the heart (48). Therefore, we argue that exercise training provides comprehensive cardioprotection, leading to the repair of cardiac function and physiological balance in hypertensive hearts.

Overall, although there is some evidence that exercise training may activate IGF and Akt signaling pathways in hypertensive animals, we argue that it is generally considered a safe and effective therapeutic intervention to improve cardiac function and reduce pathological remodeling in hypertension. It is crucial to individualize exercise training programs to the specific needs of each patient and disease stage and to monitor the intensity and duration of exercise to avoid excessive or prolonged activation of these pathways.

### 4.4. Methodological effectiveness of the included studies

In this review, we employed the CAMARADES checklist to assess the methodological effectiveness of the included studies. In the animal models, none of the included studies calculated the sample size or used blinded outcome measures. The reason is that while conducting exercise training, it is impossible to perform allocation in a blinded manner. Also, all of the studies performed a randomization of treatment or control and reported compliance with regulatory requirements, suggesting that these included studies had a proper research design and ethics. However, more than half of the included studies did not include a statement on potential conflicts of interest, while the potential for reporting bias should be a matter of concern in this review. However, all included studies were published in peer-reviewed journals with credible protocols for checking and reviewing the manuscript before publication. Of note, two studies by different authors implemented a similar exercise protocol with a minor sample size difference, obtaining different outcomes in the assessment of survival and apoptosis pathways (16, 30). Thus, the issue of bias in those study reports may be of only moderate but still reasonable concern. In addition, due to the lack of data and the concerns mentioned above, further research is needed to support our review results.

### 4.5. Limitations

In our review, some limitations can be described as follows: (1) We only extracted studies written in the English language; thus, research reports in other languages were disregarded. In addition, conference papers, unpublished papers, or locally published papers were not included. (2) There was a difference in the sex of the animal models, with six studies using male animal models and five using female animal models. In mice, it was clear that the female models showed a better response to increasing exercise capacity and hypertrophic signaling within cardiomyocytes during exercise training than the male models (54). Thus, this difference has limited the generalizability of our review to the entire population affected by hypertension. (3) The studies used different durations (5–24 weeks) with different intensity ranges of (10–27 m/min). Furthermore, almost all studies did not compare the duration and intensity of exercise training on the survival, apoptosis, and fibrosis pathways in cardiac tissue in hypertension. Thus, this review cannot provide current evidence on the optimal protocol for exercise training to repair cardiac tissue in hypertension.

## 5. Conclusions

Our systematic review provided a summary of the recent studies using animal models of hypertension to assess the effects of exercise training on cell survival, apoptosis, and fibrosis in cardiac tissue. Of the included studies, the spontaneously hypertensive rat (SHR) model and various exercise training protocols demonstrated a positive effect on hypertension in cardiac tissue. In conclusion, this review found that exercise training could elevate cell survival rates by increasing the production of IGF-1, Akt, PI3K, Bcl 2 (anti-apoptotic), and HSP 72. Furthermore, exercise training could attenuate the level of cell apoptosis in the heart by decreasing the levels of the Fas ligand, TNF, TNF receptor 1, FADD, t-Bid, Bad, Bak, Bax, caspase, and cytochrome-*c* in the cytosol. Exercise training has also been proven to decrease MAPK p38 and PTEN levels and change the physiological characteristics by normalizing the percentage of cardiac fibrosis, wall thickness, and collagen volume fraction in the left ventricle of cardiac tissue. Therefore, our systematic review suggests that exercise training may act as a therapeutic and preventive treatment for hypertension.

In addition, further studies need to be conducted to investigate the effects of exercise training on different genders, because of the pronounced differences in the underlying mechanisms of the development of hypertension. Furthermore, the protocol of exercise studies should be considered to yield an optimal protocol for the treatment of hypertension. In addition, clinical trials should be conducted to clarify the therapeutic applications of different exercise interventions in hypertension.

## Data Availability

The original contributions presented in the study are included in the article; further inquiries can be directed to the corresponding authors.
